# Prognostic significance of peritoneal cytology in low-risk endometrial cancer: comparison of laparoscopic surgery and laparotomy

**DOI:** 10.1007/s10147-020-01854-z

**Published:** 2021-01-07

**Authors:** Satoe Fujiwara, Ruri Nishie, Shoko Ueda, Syunsuke Miyamoto, Shinichi Terada, Yuhei Kogata, Tomohito Tanaka, Yoshimichi Tanaka, Masahide Ohmichi

**Affiliations:** Department of Obstetrics and Gynecology, Osaka Medical College, 2-7 Daigakumachi, Takatsuki, Osaka 569-8686 Japan

**Keywords:** Endometrial cancer, Peritoneal cytology, Laparotomy, Laparoscopic surgery, Prognosis

## Abstract

**Background:**

There is uncertainty surrounding the prognostic value of peritoneal cytology in low-risk endometrial cancer, especially in laparoscopic surgery. The objective of this retrospective study is to determine the prognostic significance of positive peritoneal cytology among patients with low-risk endometrial cancer and to compare it between laparoscopic surgery and conventional laparotomy.

**Methods:**

From August 2008 to December 2019, all cases of pathologically confirmed stage IA grade 1 or 2 endometrial cancer were reviewed at Osaka Medical College. Statistical analyses used the Chi-square test and the Kaplan–Meier log rank.

**Results:**

A total of 478 patients were identified: 438 with negative peritoneal cytology (232 who underwent laparotomy and 206 who undertook laparoscopic surgery) and 40 with positive peritoneal cytology (20 who underwent laparotomy and 20 who received laparoscopic surgery). Survival was significantly worse among patients with positive peritoneal cytology compared to patients with negative peritoneal cytology. However, there was no significant difference among patients with negative or positive peritoneal cytology between laparoscopic surgery and laparotomy.

**Conclusion:**

This retrospective study suggests that, while peritoneal cytology is an independent risk factor in patients with low-risk endometrial cancer, laparoscopic surgery does not influence the survival outcome when compared to laparotomy.

## Introduction

Endometrial cancer is the sixth most commonly diagnosed cancer and the 14th leading cause of cancer death in women, with 320,000 estimated new cases and 76,000 deaths in 2012 [1]. In Japan, the number of patients with endometrial cancer has been increasing in recent years, and 9,673 patients were reported in the 2014 annual report of the Japan Society of Obstetrics and Gynecology (JSGO). Over 50% of Japanese patients with endometrial cancer had International Federation of Gynecology and Obstetrics (FIGO) stage IA disease and were treated with surgery only [2].

Historically, comprehensive surgery in endometrial cancer, including total hysterectomy, bilateral salpingo-oophorectomy, surgical staging and peritoneal cytology has been accomplished via open laparotomy [3, 4]. Recently, however, several studies have shown that laparoscopic surgery is feasible as an alternative approach to laparotomy for patients with early-stage endometrial cancer [5–8]. In 2014 in Japan, laparoscopic surgery for stage IA patients with endometrial cancer was accepted as a medical treatment under the national health insurance system, and its popularity has gradually increased as an alternative to laparotomy.

Several reports, however, have failed to show a negative correlation between positive peritoneal cytology and prognosis among patients with stage I endometrial cancer [9–11]. As a result, in 2009, FIGO removed cytology as a staging criteria from the endometrial cancer staging system [12]. However, additional retrospective studies added further evidence that positive peritoneal cytology is a negative prognostic factor in endometrial cancer [13], 14,15]. It has remained unclear, however, whether positive peritoneal cytology is a negative prognostic factor in low-grade, early-stage endometrial cancer. Additionally, most reports presented cases where only a laparotomy was performed. To our knowledge, there have been no other reports about patients with early-stage endometrial cancer where the prognostic significance of peritoneal cytology is compared between laparoscopic surgery and laparotomy.

We conducted this retrospective study to evaluate the prognostic significance of peritoneal cytology in low-grade, early-stage endometrial cancer. In addition, we elucidated the outcomes of laparoscopic surgery for patients with positive peritoneal cytology and compared them to those of laparotomy.

## Materials and methods

### Study design

We performed an observational retrospective analysis of patients who had surgery for endometrial cancer between August 2008 and December 2019 at Osaka Medical College. Approval for this study was granted by the Institutional Review Board of the Osaka Medical College.

The data were extracted from patient records at the college, including age at diagnosis, Body Mass Index, surgical procedure, peritoneal cytology, histology, preoperative imaging, date and status at last follow-up, date and sites of recurrence, and date of death if applicable.

Patients undertook a hysterectomy and bilateral salpingo-oophorectomy with a pelvic lymphadenectomy or sentinel lymph node biopsy. If the peritoneal cytology was positive, the patients underwent a subsequent omentectomy. The surgery was performed by laparoscopy or laparotomy. During laparoscopic surgery, including hysterectomy, a uterine manipulator was not utilized in all cases. Although peritoneal cytology was excluded from the new FIGO staging classification for endometrial cancer in 2009, the procedure for obtaining washings for cytologic analysis was routinely done. The inclusion criteria consisted of the following: primary endometrial cancer of endometrioid carcinoma grade 1 or 2; hysterectomy and comprehensive staging surgery suggested by FIGO 2009 stage IA, in which the tumor was confined to the uterine body with no, or less than half, myometrial invasion. A patient was excluded if she was found to have some risk factors for recurrence (eg, lymphovascular space invasion) or have synchronous carcinoma at any other site or have any suspected or confirmed extrauterine metastases in preoperative imaging or in the final pathological results, including the results of omental.

All eligible patients were classified into 4 groups as follows: Group A, patients with negative peritoneal cytology received laparotomy; Group B, patients with negative peritoneal cytology received laparoscopy; Group C, patients with positive peritoneal cytology received laparotomy; Group D, patients with positive peritoneal cytology received laparoscopy. The objectives of this retrospective study were to reveal the prognostic significance of peritoneal cytology between surgical procedures by comparing the disease-free survival and overall survival among the four groups.

### Data analysis

The Kaplan–Meier method was used to estimate the survival probabilities of patients until death from any cause and recurrence. Kaplan–Meier plots were generated while stratifying by peritoneal cytology results and surgical procedures, and log-rank tests were used to assess the difference in survival between them. Continuous variables are expressed as the median (interquartile range) or mean ± standard deviation. The Mann–Whitney *U *test was used to compare continuous variables, and Fisher’s exact test was used to compare frequencies. A Cox proportional hazard model with the relative risk (RR) and 95% confidence interval (CI) was used for the multivariate analysis. Differences with *P *values less than 0 0.05 were considered statistically significant. JMP Pro software (ver.15, SAS Inc., Tokyo, Japan) was used for the statistical analysis.

## Results

### Demographics of survey participants

The baseline characteristics of the patients are shown in Table 1. We identified 478 patients with stage IA endometrioid carcinoma grade 1 or 2 endometrial cancer who had primary surgery including washing for peritoneal cytology. There were 232 patients with a negative peritoneal cytology by laparotomy (Group A), 206 with a negative peritoneal cytology by laparoscopy (Group B), 20 with a positive peritoneal cytology by laparotomy (Group C) and 20 with a positive peritoneal cytology by laparoscopy (Group D). In total, 226 (47.3%) and 252 (52.7%) patients underwent laparotomy and laparoscopic surgery, respectively. Within the entire population, the rate of positive cytology was 8.4% (40/478). Within the laparoscopy population, the rate of positive peritoneal cytology was 8.8% (20/226) and within the laparotomy population 8.0% (20/252). The median follow-up was 54.7 months (4–148 months), and the median follow-up duration of patients who underwent laparoscopic surgery was shorter than that of laparotomy (*p* < 0.05). Except for the follow-up duration, there were no significant differences in epidemiological and clinicopathological characteristics among the four groups.

**Table 1:** The epidemiological and clinicopathological characteristics of the patients

Characteristics	Peritoneal cytology				*p*
	Negative (*n* = 438)		Positive (*n* = 40)		
	Group A Laparotomy (*n* = 232)	Group B Laparoscopy (*n* = 206)	Group C Laparotomy (*n* = 20)	Group D Laparoscopy (*n* = 20)	
Age, median (range)	56.0 (27–84)	55.5 (30–87)	56.3 (43–71)	53.2 (30–83)	0.39
Body mass index, median (range)	24.5 (16.6–41.5)	23.3 (17.3–36.0)	22.7 (21.2–28.4)	23.8 (19.5–29.6)	0.31
Follow-up duration (month), median (range)	72.1 (4–148)	41.6 (4–111)	66.7 (9–114)	29.3 (12–66)	< 0.05
Lymph node resection, *n* (%)					0.21
Sentinel LN	0 (0%)	61 (29.6%)	0 (0%)	8 (40.0%)	
PLN	232 (100%)	145 (70.4%)	20 (100%)	12 (60.0%)	
Differential of endometrioid EC, *n* (%)					0.23
Grade 1	186 (80.2%)	180 (87.4%)	20 (100%)	12 (60.0%)	
Grade 2	46 (19.8%)	36 (12.6%)	0 (0%)	8 (40.0%)	
Postoperative adjuvant therapy, *n* (%)	0 (0%)	0 (0%)	3 (15.0%)	5 (25.0%)	0.38
Chemotherapy	0 (0%)	0 (0%)	3 (15.0%)	5 (25.0%)	
Radiotherapy	0 (0%)	0 (0%)	0 (0%))	0 (0%)	
Recurrent sites, *n* (%)	*n* = 4	*n* = 3	*n* = 4	*n* = 3	< 0.05
Intra-abdominal site	2 (50.0%)	1 (33.3%)	4 (100%)	3 (100%)	
Vagina	1 (25.0%)	1 (33.3%)	1 (25.0%)	1 (33.3%)	
Pelvic	1 (25.0%)	0 (0%)	0 (0%)	0 (0%)	
Peritoneum	0 (0%)	0 (0%)	3 (75.0%)	2 (66.7%)	
Extra-abdominal site	2 (50.0%)	2 (66.7%)	0 (0%)	0 (0%)	
Liver	1 (25.0%)	0 (0%)	0 (0%)	0 (0%)	
Lung	1 (25.0%)	1 (33.3%)	0 (0%)	0 (0%)	
Nodal	0 (0%)	1 (33.3%)	0 (0%)	0 (0%)	

*Sentinel LN *sentinel lymph node biopsy, *PLN* pelvic lymph node dissection

### The prognostic significance of peritoneal cytology

**Fig.1:**
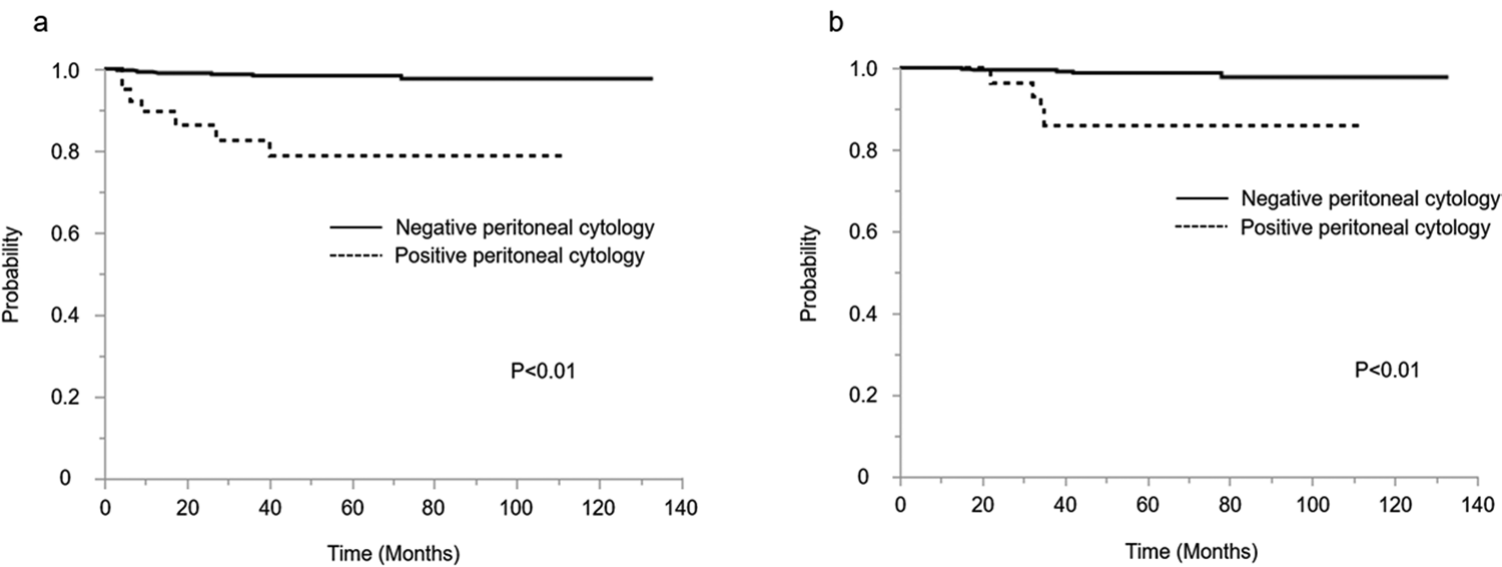
**a** Kaplan–Meier curves of 5-year disease-free survival for all patients, stratified by peritoneal cytology group. **b** Kaplan–Meier curves of 5-year overall survival for all patients, stratified by peritoneal cytology group

Within the combined laparotomy and laparoscopic surgery population, patients with positive peritoneal cytology (Group C and D) had an inferior disease-free survival and overall survival rate compared with patients with negative peritoneal cytology (Group A and B) (Fig. 1). The 5-year disease-free survival rates with positive peritoneal cytology and those with negative peritoneal cytology were 82.5% and 98.4% (*p* < 0.01), respectively. The 5-year overall survival rates with positive peritoneal cytology and those with negative peritoneal cytology were 92.5% and 99.1% (*p* < 0.01), respectively.

### Relationship between approach procedure and prognosis

**Fig.2:**
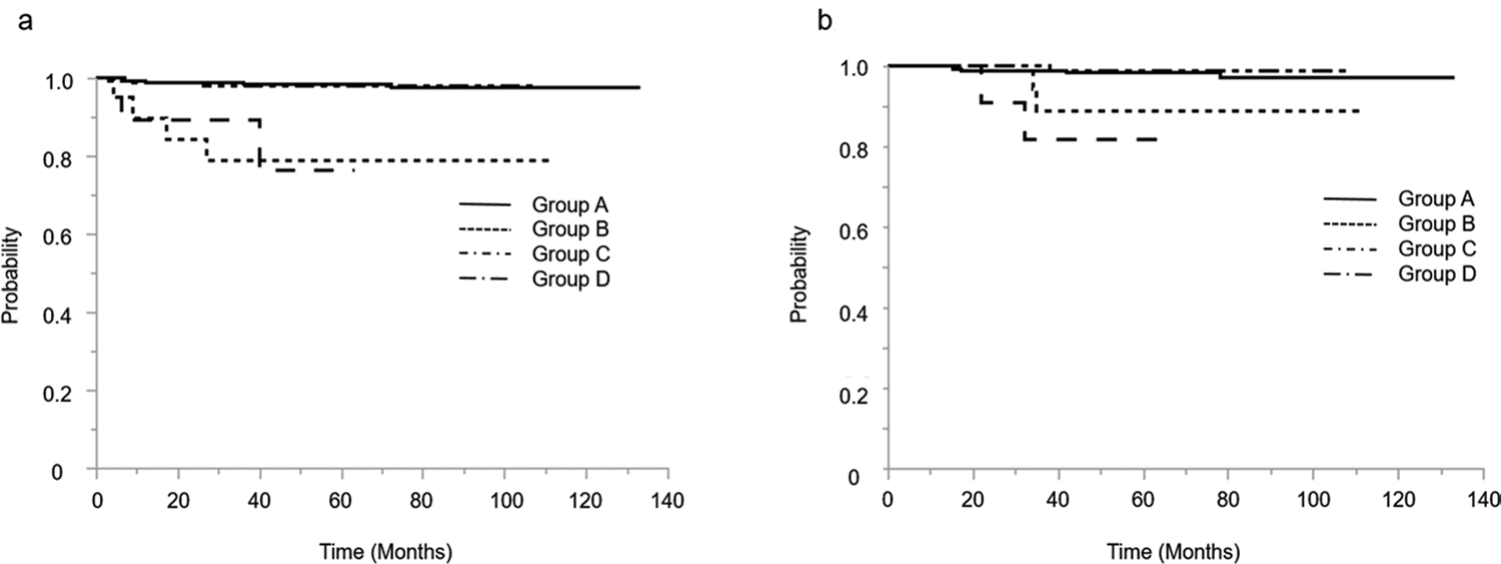
**a** Kaplan–Meier curves of 5-year disease-free survival for all patients among 4 groups. **b** Kaplan–Meier curves of 5-year overall survival for all patients among 4 groups. Group A; the patients with negative peritoneal cytology by laparotomy. Group B; the patients with negative peritoneal cytology by laparoscopy. Group C; the patients with positive peritoneal cytology by laparotomy. Group D; the patients with positive peritoneal cytology by laparoscopy

**Fig.3:**
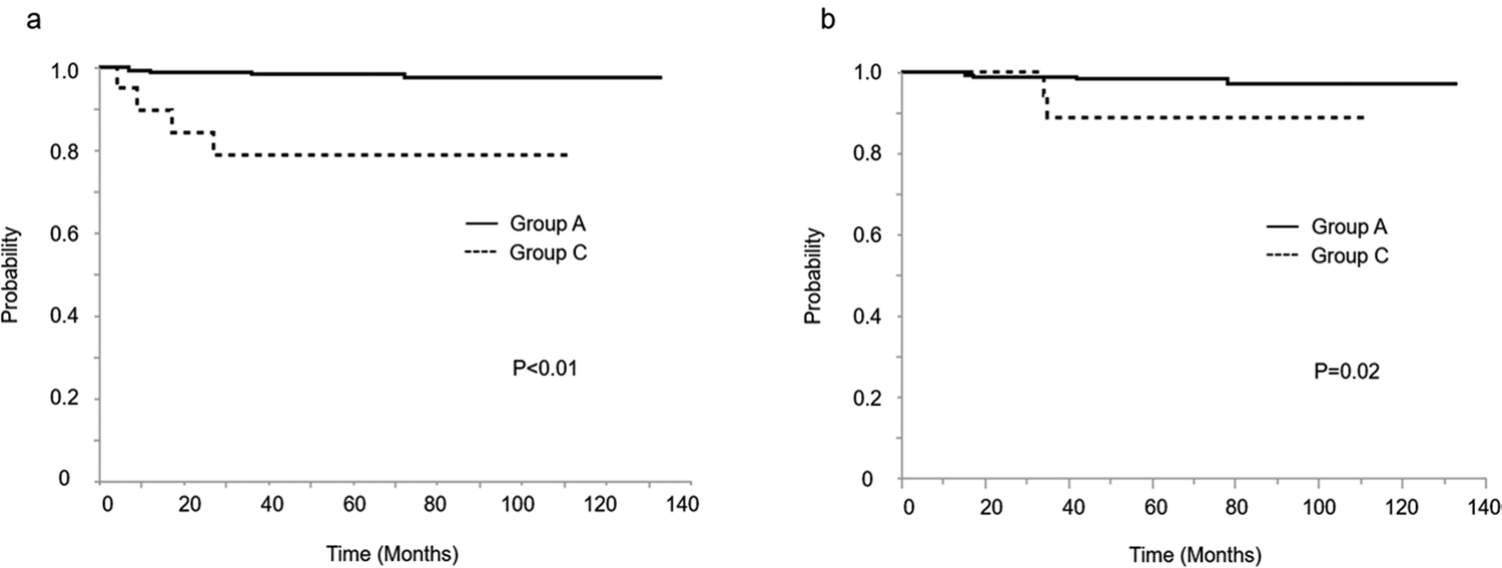
**a** Kaplan–Meier curves of 5-year disease-free survival for patients received laparotomy, stratified by peritoneal cytology. **b** Kaplan–Meier curves of 5-year overall survival for patients received laparotomy, stratified by peritoneal cytology. Group A; the patients with negative peritoneal cytology by laparotomy. Group C; the patients with positive peritoneal cytology by laparotomy

**Fig.4:**
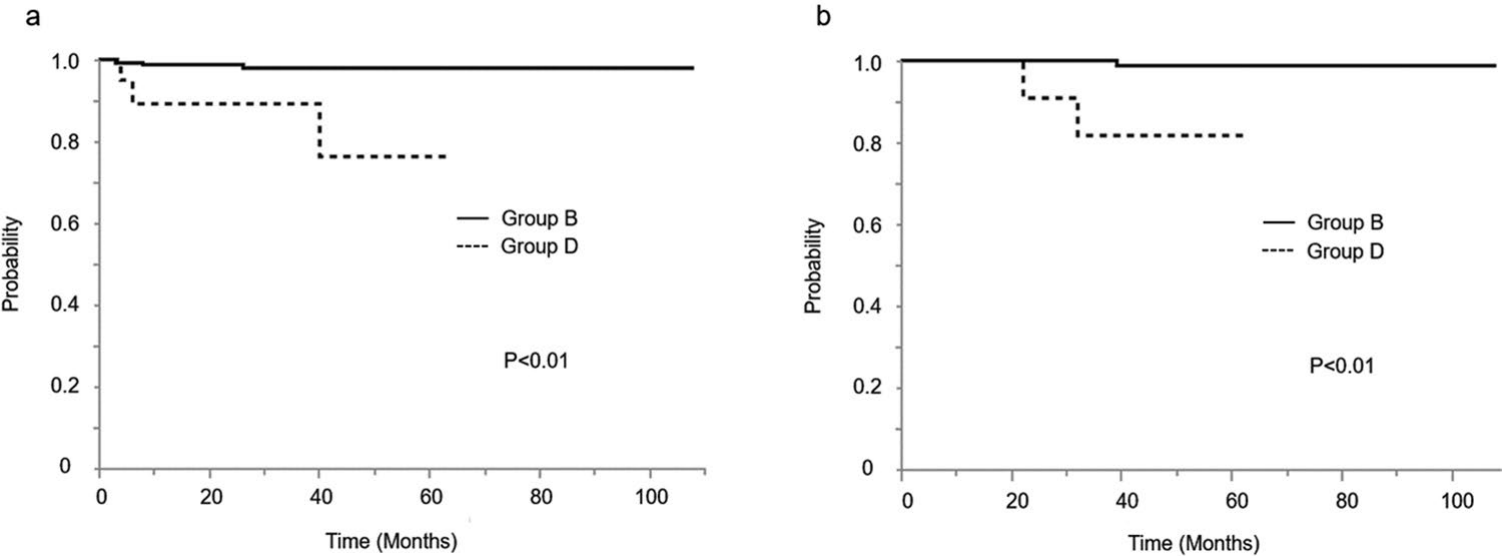
**a** Kaplan–Meier curves of 5-year disease-free survival for patients received laparoscopy, stratified by peritoneal cytology. **b** Kaplan–Meier curves of 5-year overall survival for patients received laparotomy, stratified by peritoneal cytology. Group B; the patients with negative peritoneal cytology by laparoscopy. Group D; the patients with positive peritoneal cytology by laparoscopy

The Kaplan–Meier analysis of 5-year disease-free survival and 5-year overall survival among the four groups is shown in Fig. 2. Among the patients who underwent a laparotomy, those with positive peritoneal cytology (Group C) had inferior survival rates compared to those with negative peritoneal cytology (Group A), upon more detailed examination (Fig. 3). Among the patients who received laparoscopic surgery, those with positive peritoneal cytology (Group D) also had inferior survival rates compared to those who had negative peritoneal cytology (Group B) (Fig. 4).

**Fig. 5:**
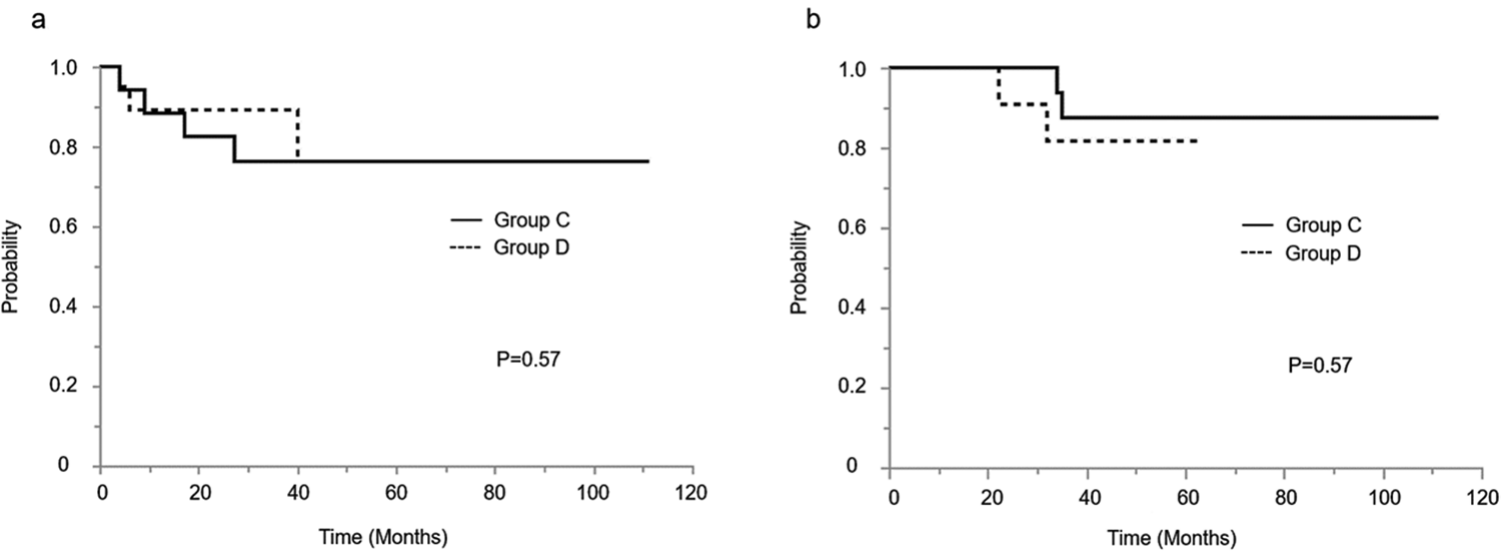
**a** Kaplan–Meier curves of 5-year disease-free survival for patients with positive peritoneal cytology, stratified by approach procedure. **b** Kaplan–Meier curves of 5-year overall survival for patients with positive peritoneal cytology, stratified by approach procedure. Group C; the patients with positive peritoneal cytology by laparotomy. Group D; the patients with positive peritoneal cytology by laparoscopy

In terms of the approach procedure, among those patients with positive peritoneal cytology, there were no significant differences between those who undertook a laparotomy (Group C) and those who received laparoscopic surgery (Group D) in both disease-free survival and overall survival (Fig. 5). The 5-year disease-free survival rates for laparotomy and laparoscopy were 80.0% and 85.0% (*p* = 0.57), respectively. Moreover, the 5-year overall survival rates for laparotomy and laparoscopic surgery were 90.0% and 95.0% (*p* = 0.57), respectively. Furthermore, Table 2 shows the results of the multivariate analysis for the progression-free survival. Patients with positive peritoneal cytology had a higher risk of disease progression than those with negative peritoneal cytology (RR [95% CI], 1.85 [1.26–2.71], *p* < 0.05). The surgical approach choice and adjuvant therapy were not associated with survival outcomes.

**Table 2:** Multivariate progression-free survival analysis

	HR (95% CI)	*p*
Differentiation (G1 ref.) G2	1.44 (0.59–3.40)	0.418
Lymph node resection (PLN ref.) Sentinel LN	1.34 (0.67–2.68)	0.408
Peritoneal cytology (negative ref.) positive	1.85 (1.26–2.71)	0.002
Adjuvant therapy (observation ref.) chemotherapy	0.69 (0.48–1.10)	0.085
Surgical procedure (laparotomy ref.) laparoscopy	1.21 (0.61–2.40)	0.583

*Sentinel LN* sentinel lymph node biopsy, *PLN* pelvic lymph node dissection, *ref*. reference

### Site of recurrence by peritoneal cytology

The site of first recurrence for the recurrences observed at the time of analysis was recorded and then retrospectively categorized into intra-abdominal (e.g., vagina, pelvic, peritoneum) and extra-abdominal (e.g., liver, lung, nodal) in Table 1. Positive peritoneal cytology increased intra-abdominal recurrence, especially intraperitoneal dissemination. In terms of the approach procedure, among those patients with positive or negative peritoneal cytology, there were no significant differences between those who undertook a laparotomy and those who received laparoscopic surgery at the site of recurrence. In the laparoscopic arm, one patient had a port-cite recurrence. It was 0.3% (1/252) of laparoscopic cases.

## Discussion

Several reports have shown no correlation between positive peritoneal cytology and survival or recurrence among patients with stage I endometrial cancer [9–11]. As a result, in 2009, the International Federation of Gynecology and Obstetrics (FIGO) removed cytology as a staging criteria from the endometrial cancer staging system [12]. However, the prognostic importance of positive peritoneal cytology in early endometrial cancer has long been debated. Garg et al. [13], Seagle et al.[14] and Shiozaki [15] reported decreased survival among patients with cytology-positive stage I/II endometrial cancer. In contrast, some reports have shown a negative prognostic association of positive peritoneal cytology among patients with stage I endometrial cancer [16, 17]. In this retrospective study, positive peritoneal cytology was associated with a decreased overall survival in patients with low-risk endometrial cancer. Those patients with low-risk endometrial cancer were defined as follows: endometrioid carcinoma grade 1 or 2, less than 50% myometrial invasion, no cervical invasion, no lymphovascular space invasion and no extra-uterine progression. In contrast to our report, those previous reports stating that positive peritoneal cytology was not associated with prognosis included cases with endometrioid carcinoma grade 3 [16, 17] and more than 50% myometrial invasion [17]. Our report suggests that positive peritoneal cytology is associated with poor prognosis in endometrial cancer patients with stage IA and endometrioid grade 1 or 2 and without any other risk factors.

We further examined the effect of positive peritoneal cytology on laparoscopic surgery in patients with endometrial cancer. There is a current trend toward laparoscopic or robotic surgery rather than surgery via laparotomy for endometrial cancer. Retrospective and prospective studies have demonstrated that the procedure is feasible in a majority of patients with stage I and thus associated with lesser complications and shorter hospitalizations[6, 18, 19]. The GOG LAP2 study was a randomized control phase III study to establish the non-inferiority of laparoscopy compared with laparotomy in terms of progression-free survival after the surgical staging of uterine cancer [6]. The LAP2 study also showed that patients with positive cytology or high-risk histology, like uterine serous carcinoma or uterine clear cell carcinoma, had poorer outcomes than patients with negative peritoneal cytology or grade 3 endometrioid carcinoma [20]. This study also revealed that there was no difference in prognosis between laparoscopic surgery and laparotomy among those patients with high-grade uterine cancer. However, there has been no other report about the prognosis between laparoscopic surgery and laparotomy among patients with positive peritoneal cytology. In our report, among patients with positive peritoneal cytology, the 5-year disease-free survival rates by laparotomy and laparoscopic surgery were 80.0% and 85.0% (*p* = 0.86), respectively. Among them, the 5-year overall survival rates for laparotomy and laparoscopy were 90.0% and 95.0% (*p* = 0.35), respectively. There were no significant differences between those patients who underwent a laparotomy and those who undertook laparoscopic surgery in both disease-free survival and overall survival. The pattern of recurrence was not significantly different between laparoscopy and laparotomy in patients with positive or peritoneal cytology. However, the incidence of intraperitoneal dissemination was more frequent in patients with positive peritoneal cytology than that in patients with negative peritoneal cytology. This event was also not significantly different between laparoscopy and laparotomy, and this result showed that positive peritoneal cytology increased intra-abdominal dissemination compared to negative cases.

Sonoda et al. reported in 2001 that the treatment of low-risk endometrial cancer by laparoscopic surgery is associated with a significantly higher incidence of subsequent positive peritoneal cytology [21]. In our study, 20 (8.8%) of those patients treated by laparoscopic surgery had positive peritoneal cytology, compared to 20 (7.9%) of those patients treated via laparotomy (*p* = 0.3). The rate of positive peritoneal cytology in patients who underwent laparoscopic surgery was similar to that of laparotomy. Our procedure was performed as follows. The uterine manipulator was not used during laparoscopic surgery. The fallopian tubes were clipped at the commencement of the operation. The specimens were retrieved in a bag to prevent the scatter of tumor cells into the peritoneal cavity. In contrast to our procedure, in the previous study, a uterine manipulator was used. Under laparoscopic assistance, there is the possibility of retrograde dissemination of cancer cells into the peritoneal cavity during uterine manipulation. Our report showed that this is not related to the rate of positive peritoneal cytology, even when comparing laparoscopic surgery and laparotomy.

In addition, our study showed that only positive peritoneal cytology impacts poor prognosis also in multivariate analysis. This result suggests that patients with positive peritoneal cytology may require adjuvant therapy. However, adjuvant chemotherapy had no effect on the survival outcomes of patients with positive peritoneal cytology in our study. However, we cannot conclude the efficacy of adjuvant therapy for those patients with positive peritoneal cytology because the number of subjects was so small. Seagle et al. [14] performed an analysis for the potential benefit of chemotherapy as an adjuvant treatment for positive peritoneal cytology. Their results suggested that treating women with positive peritoneal cytology, presumed to represent microscopic metastasis, may be associated with a survival benefit in their retrospective cohort study. That adjuvant chemotherapy may be of some benefit in this setting is strongly hypothetical, and these finds require a prospective randomized study.

Our analysis was limited because it was a retrospective study, and the number of subjects was small. Additional limitations of this study include the fact that complete data for some variables, including tumor size, were not available. However, there is no other previous report comparing laparoscopic surgery with laparotomy among patients with early low-grade endometrial cancer and positive peritoneal cytology, and our report suggests the safety of laparoscopic surgery in such cases.

In conclusion, positive peritoneal cytology was associated with the poor prognosis of patients with low-risk endometrial cancer. In addition, however, laparoscopic surgery did not influence the survival outcomes, compared to laparotomy, in patients with either positive or negative peritoneal cytology.
